# Pennsylvania

**Published:** 1897-05

**Authors:** 


### CONTROL WORK.

*Pennsylvania.* Troy, Athens, Towanda, and Canton have notified all milkdealers through their local boards of health that all animals furnishing milk for the citizens of these places must be inspected by competent veterinarians and be free from tuberculosis.

